# Nomilin targets the Keap1‐Nrf2 signalling and ameliorates the development of osteoarthritis

**DOI:** 10.1111/jcmm.15484

**Published:** 2020-06-21

**Authors:** Xing‐He Xue, Ji‐Xin Xue, Wei Hu, Fang‐Ling Shi, Yang Yang

**Affiliations:** ^1^ Department of Orthopaedics The Second Affiliated Hospital and Yuying Children's Hospital of Wenzhou Medical University Wenzhou China; ^2^ Key Laboratory of Orthopaedics of Zhejiang Province Wenzhou China; ^3^ The Second School of Medicine Wenzhou Medical University Wenzhou China

**Keywords:** chondrocytes, IL‐1β, inflammation, Keap1‐Nrf2, Nomilin

## Abstract

Osteoarthritis (OA) is a long‐term and inflammatory disorder featured by cartilage erosion. Here, we describe nomilin (NOM), a triterpenoid with inflammation modulatory properties in variety of disorders. In this study, we demonstrated the latent mechanism of NOM in alleviating the progress of OA both in vitro and in vivo studies. The results showed that NOM pre‐treatment suppressed the IL‐1β–induced over‐regulation of pro‐inflammation factors, such as NO, IL‐6, PGE_2_, iNOS, TNF‐α and COX‐2. Moreover, NOM also down‐regulates the degradation of ECM induced by IL‐1β. Mechanistically, the NOM suppressed NF‐κB signalling via disassociation of Keap1‐Nrf2 in chondrocytes. Furthermore, NOM delays the disease progression in the mouse OA model. To sum up, this research indicated NOM possessed a new potential therapeutic option in osteoarthritis.

## 1 INTRODUCTION

Osteoarthritis (OA) is a prevalent senile disorder caused by joint instability worldwide.^1^ A majority of elderly people show a representative trait of OA, which is caused by articular cartilage degradation.^2^ There are a large amount of known risk factors, including gender, ageing, mechanical stress,^3^ genetics,^4^ trauma and obesity ^5^, ^6^; however, the exact underlying pathophysiology of OA is unclear. Inflammatory environments seem to play a vital part in the pathogenesis of OA.^7^ Furthermore, it has been illustrated that Il‐1β could be involved in the progress of OA.^8^ IL‐1β release motivates the production of several pro‐inflammatory mediators and catabolic factors, such as nitric oxide (NO), thrombospondin motifs (ADAMTS), prostaglandin E2 (PGE2) and matrix metalloproteinases (MMPs) which ultimately promote the extracellular matrix (ECM) degradation and the dysfunction of chondrocytes.^9^ This inhibition of IL‐1β–induced inflammatory may be a potent target for OA.

NF‐κB pathways, which can be motivated by IL‐1β, have been reported as a master regulator in the pathogenesis of OA.^10^, ^11^ Meanwhile, as an important transcription factor, nuclear factor‐erythroid 2‐related factor‐2 (Nrf2) could promote various antioxidant gene expression including haem oxygenase‐1 (HO‐1).^12^, ^13^ Activated Nrf2 disassociates with Keap1, leading to its stabilization, nuclear translocation and activation. Furthermore, an increasing amount of evidence is reported that there is a crosstalk relationship between members of the Nrf2 and NF‐ĸB pathways in the nuclei.^14^, ^15^ In addition, Nrf2‐KO mice, compared to WT mice, shows more severe cartilage erosion.^16^ Therefore, targeting Nrf2 is emerging as a potent agent in treating OA.

Nomilin (NOM) exists in some edible citrus fruits. Previous reports have demonstrated that NOM has a wide range of biological activities such as anti‐inflammatory,^17^ anticancer^18^ an antioxidant.^19^ Chen found that NOM inhibited the oxidative stress via activating Nrf2 in cerebral ischaemia‐reperfusion rats.^20^ However, the anti‐inflammatory effects of NOM in OA are still unknown. In the present research, we evaluated the anti‐inflammatory effect of NOM in IL‐1β–induced mice chondrocytes and explored the possible mechanism.

## 2 MATERIALS AND METHODS

### 2.1 Reagents and antibodies

Nomilin (purity > 98%) was purchased from Shanghai aladdin Medical Technology Co., Ltd. Recombinant human IL‐1β, dimethylsulphoxide (DMSO) and type II collagenase were purchased from Sigma‐Aldrich. The primary antibody against collagen Ⅱ, Lamin B1, iNOS, COX‐2 and GAPDH was acquired from Abcam, goat anti‐rabbit and antimouse IgG‐HRP were from Bioworld and antibodies against Keap1, Nrf2, HO‐1, COX‐2, IκBα and p65 were purchased from Cell Signaling Technology; Alexa Fluor^®^488 labelled and Alexa Fluor^®^594 labelled Goat Anti‐Rabbit IgG (H + L) second antibody was purchased from Jackson ImmunoResearch. The 4′, 6‐diamidino‐2‐phenylindole (DAPI) was obtained from Beyotime. The cell culture reagents were purchased from Gibco. Foetal bovine serum (FBS), bovine serum albumin (BSA), Dulbecco's modified Eagle's medium (DMEM)/Ham's F12 medium and 0.25% trypsin‐ethylenediaminetetraacetic acid (trypsin–EDTA) were purchased from Gibco (Life Technologies Corp.). TRIzol reagent was purchased from Invitrogen. Quanti Tect Reverse Transcription kit was purchased from Qiagen. SYBR Green Master Mix was purchased from Bio‐Rad Laboratories. ELISA kits of PGE2, TNF‐α, IL‐6, Collagen II, Aggrecan, MMP‐13 and ADAMTS‐5 were purchased from R&D systems. Griess reagent was purchased from Beyotime Institute of Biotechnology.

### 2.2 Primary mice chondrocytes culture

Ten immature C57BL/6 mice (5 males and 5 females, 3 days) were killed with an overdose of sodium pentobarbital. The knee cartilages of mice were collected carefully under aseptic conditions by a dissecting microscope, and the tissues were treated with 2 mg/mL (0.1%) collagenase II for 4 hours at 37°C. Next, the digested cartilage tissues were suspended and seeded into tissue culture flasks. The chondrocytes grow in DMEM/F12 (Gibco, Invitrogen) with 10% foetal bovine serum (FBS; Hyclone, Thermo Scientific) and 1% penicillin/streptomycin antibiotics (Gibco, Invitrogen) in the incubator maintained at 5% CO_2_ at 37°C. The medium was changed firstly after 24 hours incubation. When up to 80%‐90% confluency, the cells were harvested by using 0.25% Trypsin‐EDTA (Gibco, Invitrogen). Then, cells were replanted into 10‐cm culture plates at the appropriate density. The second‐passage chondrocytes were used for all of our experiment due to no significant changes were noticed during cells passaging from passage 0 to passage 2. The chondrocytes were cultured in the incubator maintained at 5% CO2 at 37°C, and the complete medium was changed every other day.

### 2.3 Cell viability assay

The cytotoxicity of NOM on chondrocytes was measured by the cell counting kit‐8 (CCK‐8; Dojindo Co.) according to the manufacturer's protocol. Briefly, the first‐passage chondrocytes were cultured for 24 hours in a 96‐well plates (50 000 cell/ cm^2^) and then incubated in different concentration of NOM (0, 5, 10, 20 µmol/L) for 24 hours. At the indicated time, the cells were washed with phosphate‐buffered saline (PBS), followed by addition of 100 µL of DMEM/F12 containing 10 µL of CCK‐8 solution each well of the plate and incubated for another 2 hours at 37°C. Then, the absorbance of the wells was read at 450 nm using a spectrophotometer (Thermo Fisher). All experiments were performed five times.

### 2.4 NO, PGE2, TNF‐α, IL‐6, collagen II, aggrecan, ADAMTS‐5 and MMP13 measurement

The NO level in culture medium was detected by Griess reagent. The concentration of PGE2, TNF‐α, IL‐6, collagen II, aggrecan, ADAMTS‐5 and MMP‐13 in cell culture supernatants was determined by using commercial enzyme‐linked immunosorbent assay (ELISA) kits (R&D Systems) according to the manufacturer's instructions. All assays were performed five times.

### 2.5 Western blotting

The total protein extracted from chondrocytes was isolated using RIPA lysis buffer with 1 mmol/L PMSF (phenylmethanesulphonyl fluoride) and on the ice for 10 minutes followed by 15 minutes centrifugation at 12 000 rpm and 4°C, and then, protein concentration was measured using the BCA protein assay kit (Beyotime). 40 ng of protein was separated by sodium dodecyl sulphate‐polyacrylamide gel electrophoresis (SDS‐PAGE) and transferred to a polyvinylidene difluoride membrane (Bio‐Rad, USA). After blocking with 5% non‐fat milk for 2 hours, the membranes were incubated with the primary antibody against GAPDH (1:5000), iNOS (1:1000), COX‐2 (1:1000), p65 (1:1000), IκBα (1:1000), Lamin B1 (1:1000), Keap1 (1:1000), HO‐1 (1:1000) and Nrf2 (1:1000) overnight at 4°C, and followed by subsequent incubation with respective secondary antibodies for 2h at room temperature. After 3 times washing with TBST, the blots were visualized by electrochemiluminescence plus reagent (Invitrogen). Finally, the intensity of these blots was quantified with Image Lab 3.0 software (Bio‐Rad).

### 2.6 siRNA transfection

Double‐stranded siRNA for human Nrf2 gene silencing was designed and chemically synthesized (RiboBio), and the sequences of the Nrf2 siRNA were as follows: sense strand 5′‐GUAAGAAGCCAGAUGUUAADUDU‐3′. Nrf2 and negative control of siRNA transfection were undertaken using Lipofectamine 3000 siRNA transfection reagent (Thermo Fisher).

### 2.7 Immunofluorescence

The immunofluorescence staining was performed in a vitro study. Chondrocytes were divided into the following three groups: (a) control group, (b) IL‐1β–stimulated group and (c) IL‐1β plus NOM (10 µmol/L)‐treated group. For collagen II staining, the chondrocytes were planted in glass plates in a six‐well plate, and then, the cells were treated with 10 ng/mL IL‐1β or being co‐treated with 10 ng/mL IL‐1β and 10 µmol/L NOM for 24 hours in medium after incubated with serum‐starved medium overnights. For p65 staining, the duration of IL‐1β and NOM treatment was down to 2 hours. After treatments. The samples were rinsed three times in PBS before fixation using 4% paraformaldehyde and followed by permeation using the 0.1% Triton X‐100 diluted in PBS for 15 minutes. Then, the cells were blocked with 5% bovine serum albumin for 1 hour at 37°C, rinsed with PBS and incubated with primary antibodies which diluted in PBS: collagen Ⅱ (1:200), MMP13 (1:200), Nrf2 (1:200) and p65 (1:200) in a humid chamber overnight at 4°C. On the next day, the glass plates were washed and incubated with Alexa Fluor^®^488 labelled or Alexa Fluor^®^594 conjugated second antibodies (1:400) for 1 hour at room temperature and labelled with DAPI for 5 minutes. Finally, five fields of each slides were chosen randomly for microscopic observation with a fluorescence microscope (Olympus Inc.), and the fluorescence intensity was measured using ImageJ software 2.1 by observers who were blinded to the experimental groups.

### 2.8 Immunoprecipitation

Following treatments, cell lysates were prepared and incubated with sufficient amount of anti‐Keap1 antibody for 1 hour. The immune complexes were retrieved with protein G Sepharose beads at 4°C overnight. The precipitates were washed four times with ice‐cold PBS, proteins were then released by boiling in sample buffer, and the Keap1 and Nrf2 levels were further detected by immunoblotting using the anti‐Keap1 and anti‐Nrf2 antibody (IB) as described above.

### 2.9 Animal experiments

Fifteen‐week‐old C57BL/6 male wild‐type (WT) mice were obtained from the Animal Center of Chinese Academy of Sciences, Shanghai, China. Care and use of all animals conformed to the Guidelines set forth by the Chinese National Institutes of Health, with relevant study protocols also approved by the Animal care and Use Committee of Wenzhou Medical University. OA induction was performed as previously described.^21^ In this study, mice were randomly divided into three groups of 15 mice to establish a sham control group (sham), an osteoarthritis group (OA) and an osteoarthritis treated with NOM group (NOM). NOM was dissolved in 0.5% carboxymethylcellulose sodium to form oral suspension. Mice in sham group were made by sham operation. Mice in NOM group received a gavage of NOM (20 mg/kg/d) for 8 weeks after surgery while mice in OA group received a gavage of 0.5% carboxymethylcellulose sodium.

### 2.10 X‐ray imaging method

After 8 weeks of surgery with or not without treatment, the animals were given the X‐ray examination. X‐ray imaging was performed on all mice to evaluate the joint space, osteophyte formation and calcification changes of cartilage surface using a digital X‐ray machine (Kubtec Model XPERT.8; KUB Technologies Inc.). Proper images were obtained in the following settings: 50 Kv and 160 µA.

### 2.11 Histological analysis

Knee joints were fixed in 4% paraformaldehyde for 24 hours at 4°C and decalcified in 10% EDTA solution at 4°C for 4 weeks. The specimens then were embedded in paraffin and cut into 5 μm thick sections. The sections were stained with haematoxylin and Eosin (H&E) and Safranin O‐Fast Green staining to assess cartilage condition. The histological assessment was performed according to the grading of Osteoarthritis Research Society International (OARSI) scoring system in a blinded manner. We applied AxioVision software to measure the thickness of the medial subchondral bone plate according to Safranin O stained sections.

### 2.12 Statistical analysis

Data are expressed as means ± standard deviation (SD) of the representative experiment performed in triplicate. One‐way ANOVA analysis was used for the statistical comparison of multiple groups. Non‐parametric data (like OARSI score) were analysed by the Kruskal‐Wallis H test. *P* < .05 was considered statistically significant.

## 3 RESULTS

### 3.1 Cell viability

**Figure 1:**
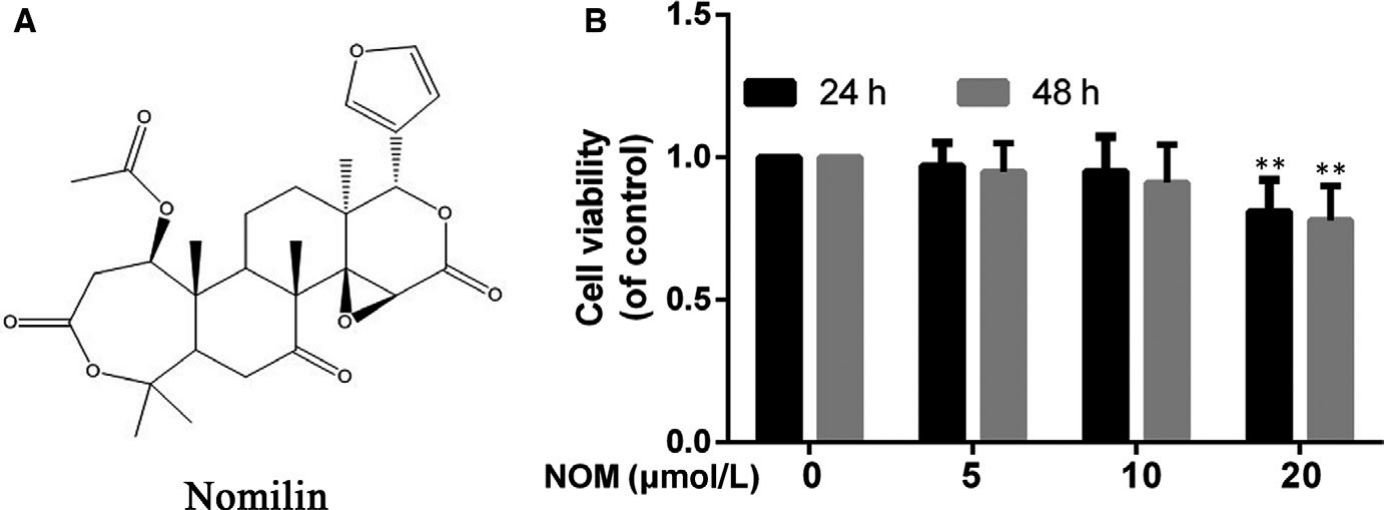
Effect of NOM on mice osteoarthritis (OA) chondrocyte viability. A, Chemical structure of NOM. B, Cells were treated with different concentrations of NOM (5, 10 and 20 μmol/L) for 24 or 48 h and analysed using a CCK‐8 assay. Data are expressed as mean ± SD. All experiments were repeated three times. *P < .05 compared with control group

Figure 1A showed the chemical formula of NOM. The cytotoxic effect of NOM (0, 5, 10 and 20 µmol/L) was assessed by Cell Counting Kit‐8 (CCK8) assay. CCK8 results, showed in Figure 1B, illustrated that NOM was cytotoxic to mice chondrocytes at 20 µmol/L after 24 and 48 hours, but cell viability was not affected by NOM (≤10 µmol/L). Thus, the concentrations of NOM (5 and 10 µmol/L) were used in subsequent experiments.

### 3.2 NOM ameliorates IL‐1β–stimulated mice chondrocytes on expression of iNOS, COX‐2, NO, PGE2, IL‐6 and TNF‐α

**Figure 2:**
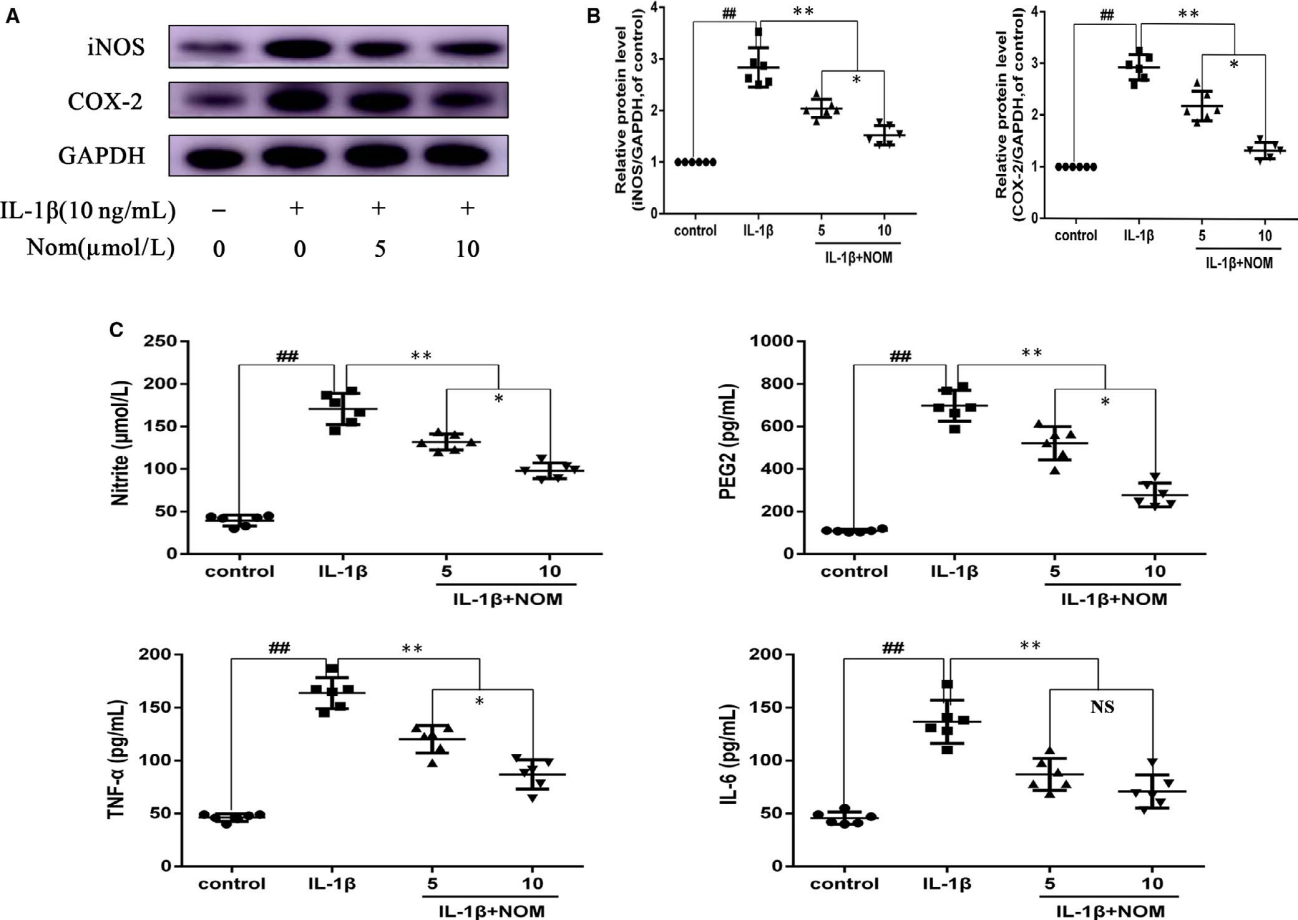
Effect of NOM on IL‐1β–induced inflammatory response in mice chondrocytes. Chondrocytes were pre‐treated for 2 h with various concentrations of NOM (5 and 10 μmol/L) and then stimulated with or without stimulated with IL‐1β (10 ng/mL) for 24 h. The protein expression of iNOS and COX‐2 assessed by Western blot and quantification analysis (A, B). Effect of NOM on IL‐1β–induced PGE2, NO, TNF‐α and IL‐6 production in mice chondrocytes (C). Data are expressed as mean ± SD. All experiments were repeated five times. **^##^** *P* < .01 compared with control group. **P < .01 compared with IL‐1β group. *P < .05 compared with NOM (5 μmol/L) group. NS compared with NOM (5 μmol/L) group

Chondrocytes pre‐treated with NOM with 5 and 10 μmol/L for 2 hours suppressed the up‐regulation of iNOS and COX‐2 stimulated by IL‐1β (Figure 2A,B). Moreover, as shown in Figure 2C, the pre‐treatment of NOM reduced NO generation and PGE2 expression concentration‐dependently. Additionally, as shown in Figure 2C, NOM inhibited IL‐1β–induced production of TNF‐α and IL‐6 production, respectively. Altogether, these data illustrated that NOM suppressed the secretion of inflammatory mediators.

### 3.3 NOM suppressed ECM deterioration on IL‐1β–stimulated mice chondrocytes

**Figure 3:**
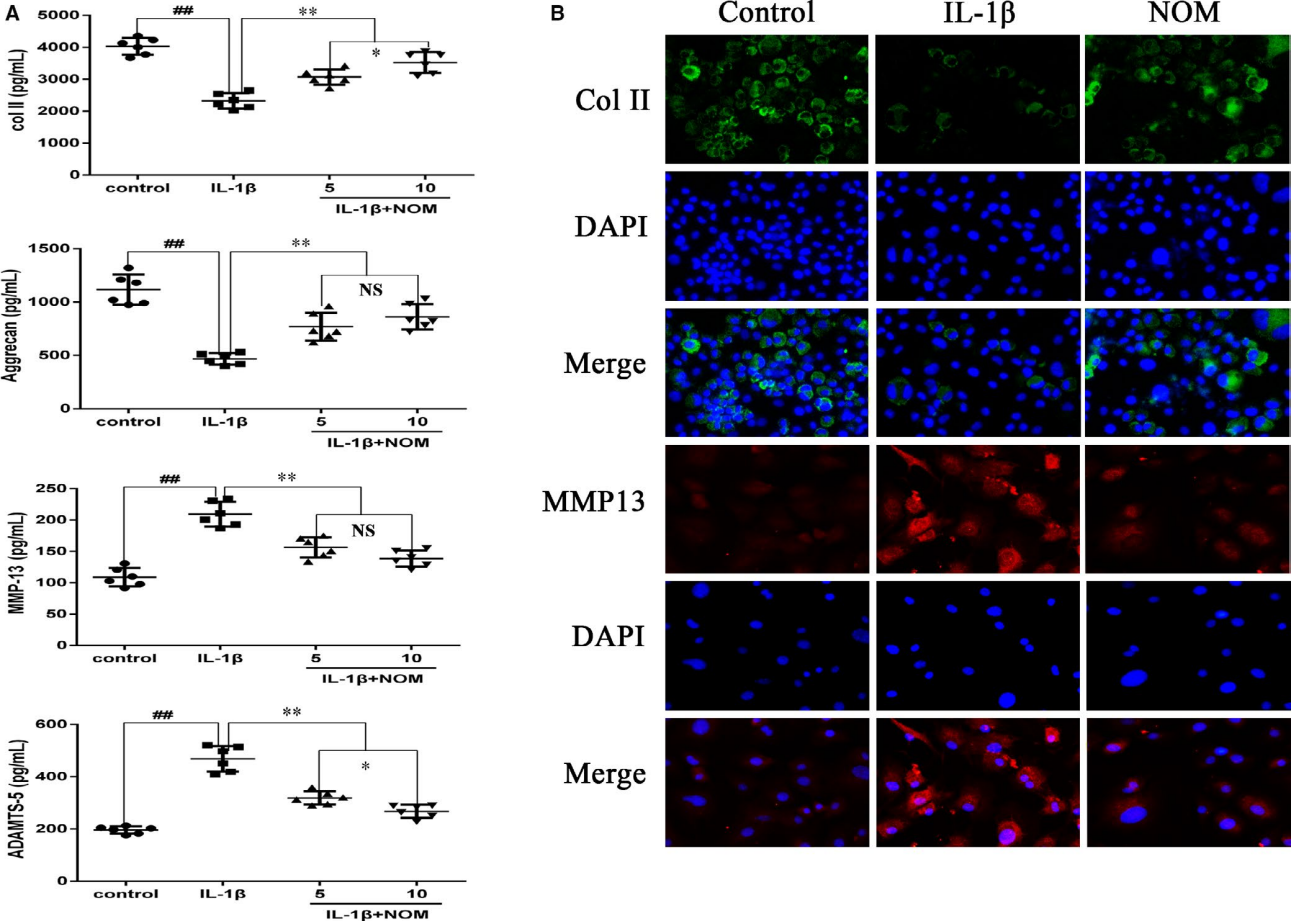
Effect of NOM on extracellular matrix degradation from IL‐1β–induced mice chondrocytes. Chondrocytes were pre‐treated with various concentrations of NOM (0, 5 and 10 μmol/L) for 2 h, followed by stimulation with or without IL‐1β (10 ng/mL) for 24 h. The protein expressions of type II collagen, aggrecan, MMP‐13 and ADAMTS‐5 in chondrocytes were visualized by ELISA (A). The representative type II collagen (Col II) and MMP13 (B) were detected by the immunofluorescence combined with DAPI staining for nuclei (original magnification × 200, scale bar: 50 µm). The values are mean ± SD of five independent experiments. ^##^ *P* < .01 compared with control group. **P < .01 compared with IL‐1β group. *P < .05 compared with NOM (5 μmol/L) group. NS compared with NOM (5 μmol/L) group

We next examined the protective influence of NOM on collagen II and aggrecan degradation. As shown in Figure 3A, NOM promoted collagen II and aggrecan synthesis, and suppressed the production of ADAMTS5 and MMP13 in mice chondrocytes. All changes by IL‐1β‐induced were suppressed by pre‐treatment with NOM, especially at the concentration 10 µmol/L. Meanwhile, the results of immunofluorescence staining were consistent with the ELISA results (Figure 3B).

### 3.4 NOM suppressed NFκB activation on IL‐1β‐stimulated mice chondrocytes

**Figure 4:**
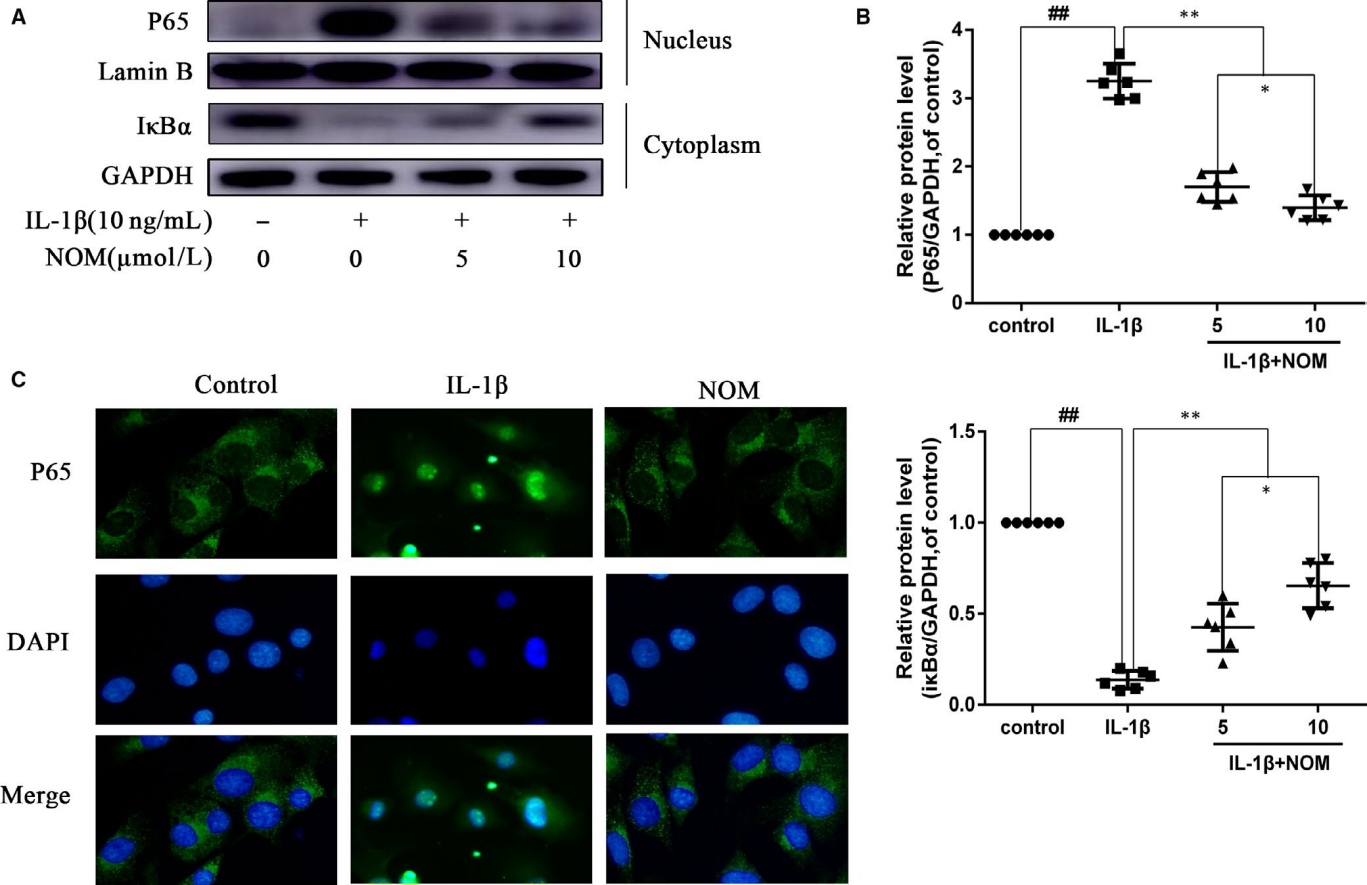
Effect of NOM on IL‐1β–induced NF‐κB activation. Chondrocytes were pre‐treated with various concentrations of NOM (0, 5 and 10 μmol/L) for 2 h, followed by stimulation with or without IL‐1β (10 ng/mL) for 1 h. The protein expressions of IκBα in cytoplasm and p65 in nuclear in chondrocytes were visualized by Western blotting (A) and quantified in (B). The nuclei translocation of p65 was detected by the immunofluorescence combined with DAPI staining for nuclei (C, original magnification × 400, scale bar: 10 µm). The values are mean ± SD of five independent experiments. ^##^ *P* < .01 compared with control group. **P < .01 compared with IL‐1β group. *P < .05 compared with NOM (5 μmol/L) group. NS compared with NOM (5 μmol/L) group

NF‐κB plays a crucial part in IL‐1β‐stimulated inflammation. Therefore, we performed Western blot analysis to scrutinize alterations in the NF‐κB signalling. As illustrated in Figure 4 A, B, IL‐1β motivated IκBα deterioration and up‐regulated the p65 in the nucleus at protein level. In contrast, these alterations were inhibited by NOM pre‐treatment. As shown in Figure 4C, under the treatment of IL‐1β, P65‐active proteins were observed in the nucleus. However, pre‐treating with NOM inhibited the translocation of P65 brought by IL‐1β stimulation. These results are in keeping with suppression of the NF‐κB P65 pathway detected by Western blot.

### 3.5 NOM suppressed Keap1‐Nrf2 pathway on IL‐1β–stimulated mice chondrocytes

**Figure 5:**
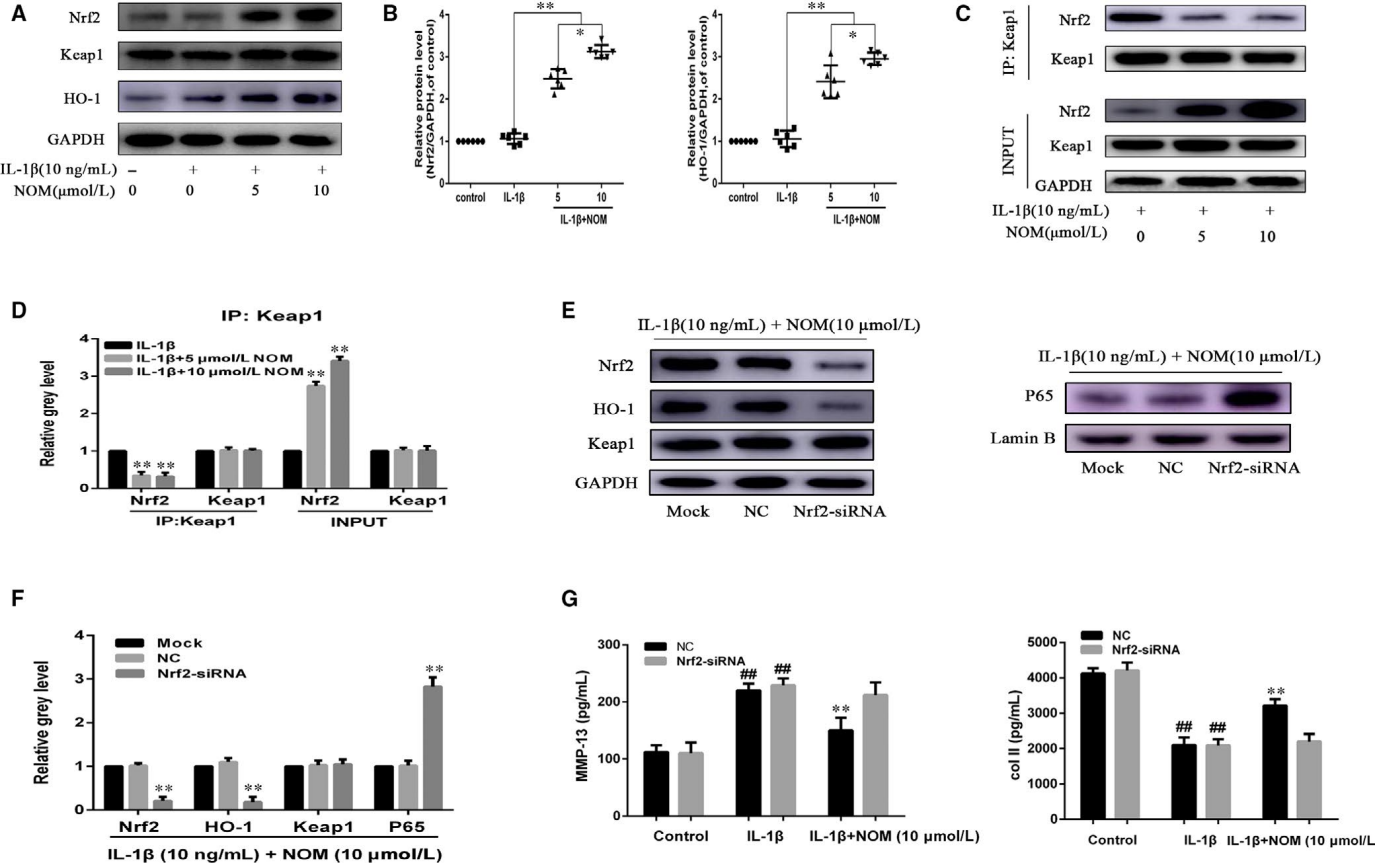
Effect of NOM on Keap1‐Nrf2 pathway. Chondrocytes were pre‐treated for 1 h with various concentrations of NOM (0, 5 and 10 μmol/L), followed by stimulation with or without IL‐1β (10 ng/mL) for 2 h. Nrf2, Keap1 and HO‐1 were determined by Western blot (A) and quantification analysis (B). Total proteins extracted were subjected to immunoprecipitation (IP) using specific antibodies against Keap1. Immunoprecipitates were then subjected to Western blot analyses using specific antibodies against Keap1 and Nrf2 (C). Quantitative analyses of immunoprecipitates relative to GAPDH by densitometry were conducted (D). After Nrf2 knock‐down, the protein expressions of Nrf2, HO‐1 and P65 in chondrocytes treated as above were visualized by Western blot (E) and quantified in (F). The production of MMP‐13 and collagen II was assessed by ELISA (G). The values are mean ± SD of five independent experiments. **P < .01 compared with IL‐1β group. *P < .05 compared with NOM (5 μmol/L) group. NS compared with NOM (5 μmol/L) group

Pre‐treating chondrocytes with NOM activated Nrf2 expression in a dose‐dependent pattern (Figure 5A,B). What's more, co‐immunoprecipitation (co‐IP) results illustrated that Nrf2 combined with Keap1 in IL‐1β–induced mice chondrocytes. However, NOM pre‐treatment inhibited Keap1‐Nrf2 combination (Figure 5C,D, IP), making Nrf2 protein stabilization (Figure 5C,D, Input). Furthermore, our data illustrated that Nrf2 siRNA transfection down‐regulated Nrf2/HO‐1 expression in NOM pre‐treated under inflammatory condition (Figure 5E,F). Moreover, expression of P65 was increased after Nrf2 siRNA transfection, which reveals that Nrf2 mediated the anti‐inflammation of NOM in mice chondrocytes. Furthermore, we examined the MMP‐13 and type II collagen expression by ELISA assay. Our data illustrated that Nrf2 siRNA abolished the NOM‐treated inhibition of ECM deterioration under the IL‐1β treatment (Figure 5G).

### 3.6 NOM ameliorates OA development in the mouse model of DMM

**Figure 6:**
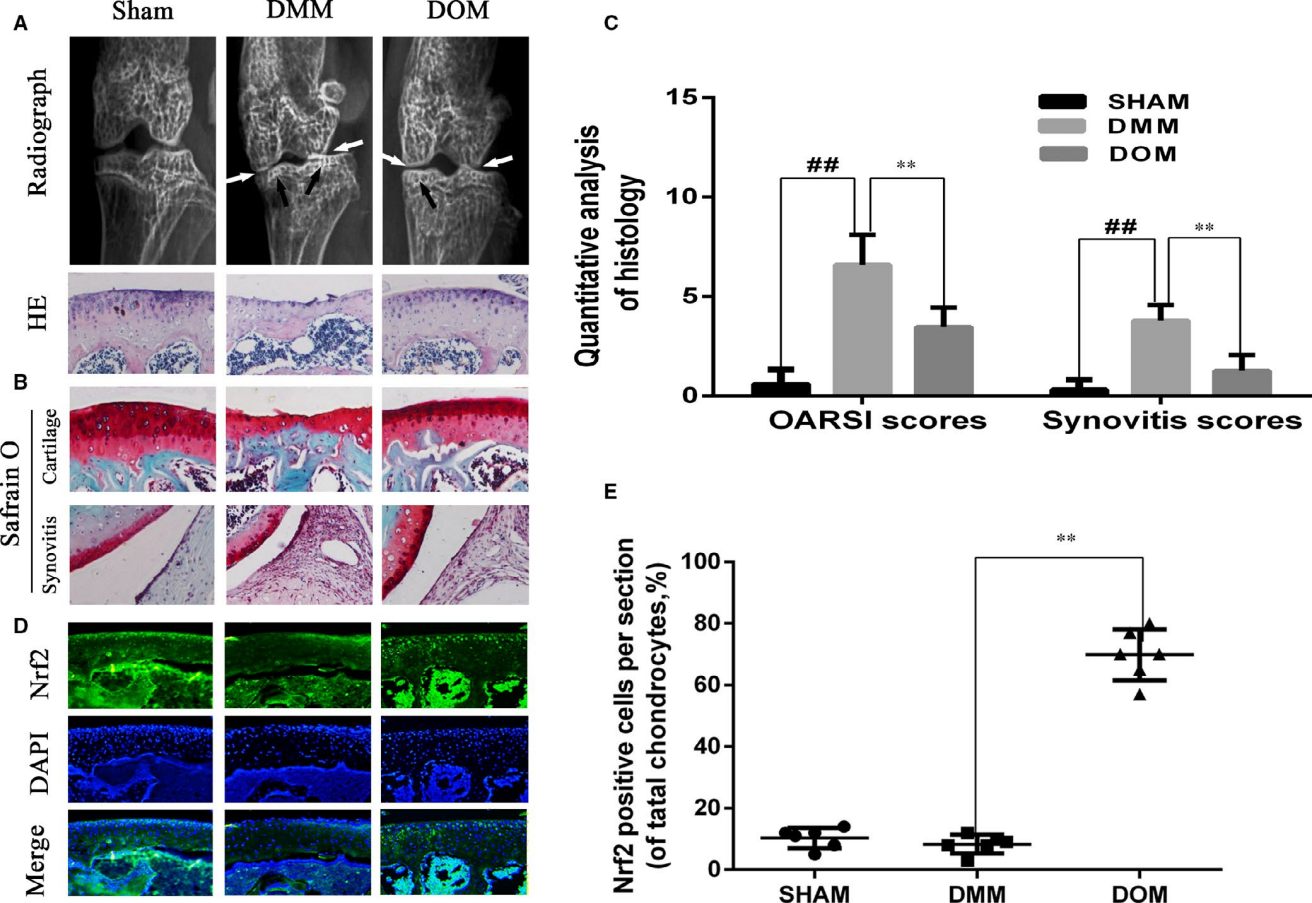
Nomilin alleviated the progression of osteoarthritis (OA) in mice OA models. Mice received a gavage of NOM or vehicle (carboxymethylcellulose sodium) 14 d after surgery. Histological analysis of OA was evaluated by haematoxylin‐eosin stainin (A) and Safranin O staining (B). Osteoarthritis Research Society International (OARSI) scores and Synovitis scores (C) were calculated for each group. Immunofluorescence staining of Nrf2 expression in the cartilage samples (D). The percentage of nuclear Nrf2‐positive cells of total chondrocytes each sections (E). Data are mean ± SD. ##*P* < .5 compared with control group, **P < .05 compared with OA group

To study whether NOM has protective impact on OA development in vivo *experiments*, surgical OA model was developed in mice, followed by peroral treatment of 20 mg/kg NOM daily for 14 days. According to X‐rays, the joint space was severe narrowed and osteosclerosis was increased after surgery. Nevertheless, lower narrow of joint gap and milder calcification of joint surface was perceived in NOM group. Histological analysis was determined by Safranin O/Fast Green staining and haematoxylin‐eosin staining. Synovitis scores and OARSI scores were used for quantitative study. As shown in Figure 6 A‐C, the joint surface was red staining with smooth cartilage surface in the control group, while the DMM group illustrated cartilage cauterization and massive proteoglycan degradation. NOM had a smoother surface of joint. Consistent with these data, the OARSI scores and Synovitis scores in the NOM group were inferior than the OA group. In addition, the Nrf2‐positive chondrocytes are increased after NOM treatment (Figure 6 D,E). Taken together, these results demonstrated that NOM ameliorates OA progression relates to the Nrf2 pathway in animal experiment.

## 4 DISCUSSION

Osteoarthritis is one of the most widespread ageing‐related joint disorders in elder adults.^22^ The molecule mechanisms of OA remain unknown and without any effective agent. Currently, common OA treatment is major in pain relief and surgery for terminal stage of OA.^23^, ^24^ Although multiple non‐surgical regimens are widely applied in OA therapy, these drugs only relieve OA symptoms and are restricted for many aspects including limited efficacy.^25^ Thus, a more effective agent is in urgent need.^26^ NOM, isolated from common edible citrus fruits, serves as a traditional medicine that has been reported relevant to a large range of anti‐inflammatory activities in numerous inflammation‐associated disorders.^27^ However, its protectiveness and exact mechanisms involved in degenerative diseases remain unknown. In this study, we evaluated whether NOM was protective against IL‐1β–stimulated inflammation and ECM metabolism in OA.

Previous studies confirmed that NF‐κB pathway is a catabolic pathway refer to the regulation of ECM equilibrium associated with the pathogenesis of OA.^10^, ^28^ During the IL‐1β–stimulated inflammation, IL‐1β promotes IκB phosphorylation and degradation, stimulating translocation of P65. P65 takes part in the transcription of pro‐inflammatory mediators and catabolic enzymes. Among these factors, iNOS catalyses NO, which impede ECM synthesis and promote the ECM deterioration.^29^ As a member of inflammation, PGE2 is generated from COX‐2 and also promotes the secretion of ADAMTS5 and MMPs.^30^ MMP13 is a subgroup of collagenases member that play a principal part in the catabolism of ECM, and ADAMTS5 leads to the cleavage of aggrecan.^31^ In this research, NOM inhibited the overproduction of PGE2, NO, TNF‐α and IL‐6 as well as the overexpression of COX‐2 and iNOS. Moreover, similar results were also found for ECM metabolism in OA. In addition, our data showed that NOM suppresses IL‐1β–stimulated inflammation via inhibition of NF‐κB signalling in mice chondrocyte.

It is reported that Nrf2 pathway is considered a target for inflammation stimulated by anti‐NF‐κB.^12^ In the unstimulated condition, Nrf2 normally binds to Keap1 results in Nrf2 sequestration and deterioration.^32^, ^33^ Motivated Nrf2 separates from Keap1, turns into nucleus and later combines with antioxidant‐responsive elements (ARE) to stimulate transcription of numerous antioxidant proteins including HO‐1. Previous studies showed that Nrf2 and HO‐1 inhibit inflammation via the suppression of P65 translocation.^12^ Activated Nrf2 could retard the progression of OA.^14^ We found that NOM stimulated Keap1‐Nrf2 disaggregation and Nrf2 protein translocation, promoting transcription of numerous known Nrf2 target genes.

**Figure 7:**
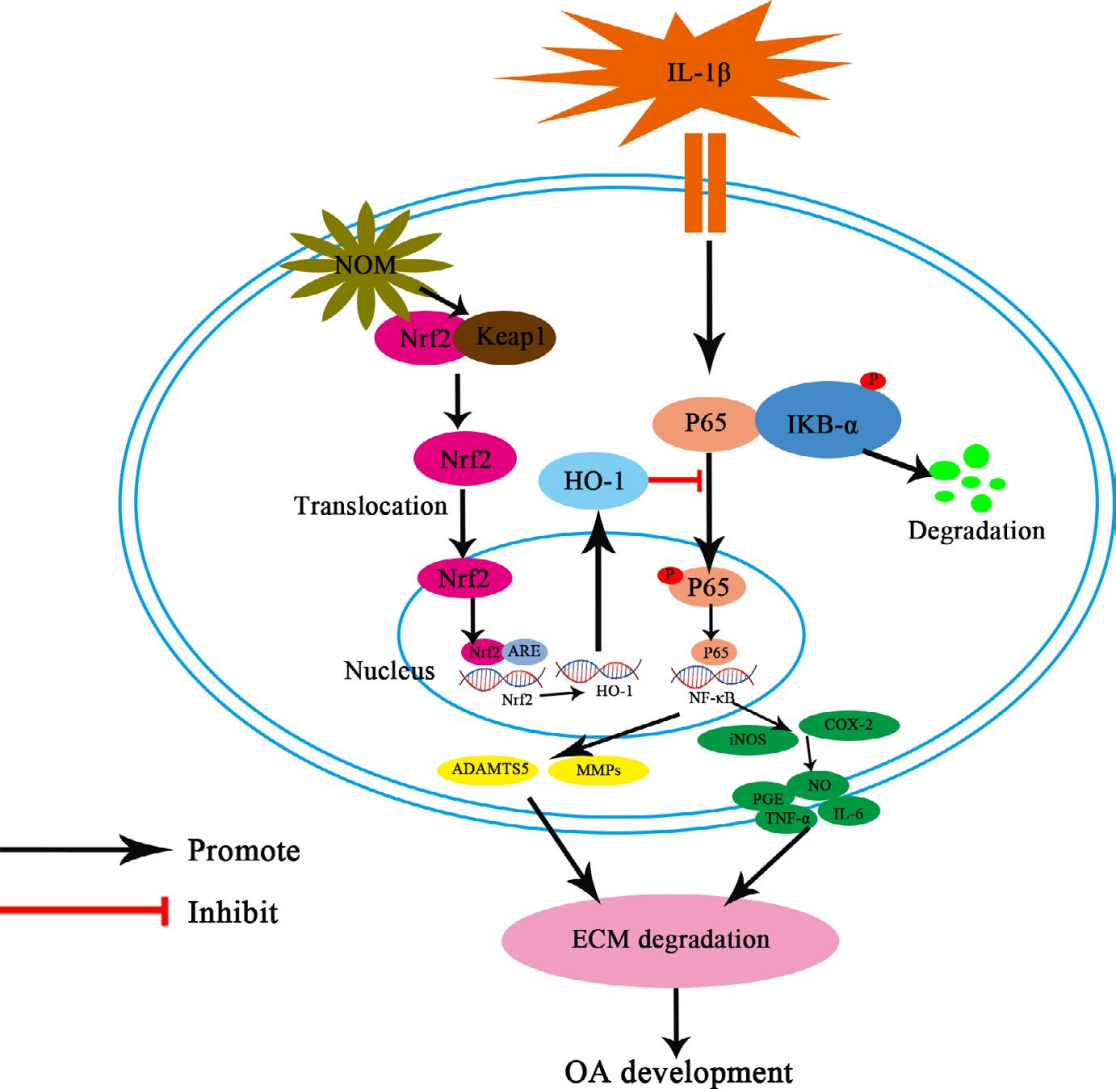
Working model for the inhibitory of NOM on IL‐1β–induced inflammation and ECM degradation in mice chondrocytes by targeting the Keap1‐Nrf2 signalling. ECM, extracellular matrix; HO‐1, haem oxygenase‐1; IL, interleukin; Keap1, Kelch‐like ECH‐associated protein 1; NF‐κB, nuclear factor κB; NOM, nomilin; Nrf2, nuclear factor‐erythroid 2‐related factor‐2; OA, osteoarthritis

Taken together, We demonstrate that NOM impeded the IL‐1β–stimulated inflammation via blocking the activation of NF‐κB signalling via disassociation of Keap1‐Nrf2 in chondrocyte (Figure 7). All these data suggest the use of NOM as a promising regimen in the treatment of OA.

## CONFLICT OF INTEREST

The authors declare that they have no conflict of interest.

## AUTHOR CONTRIBUTIONS

**Xing‐He Xue:** Data curation (lead); Funding acquisition (equal); Writing–original draft (equal). **Ji‐Xin Xue:** Methodology (equal); Writing–original draft (equal). **Wei Hu:** Investigation (equal); Writing–review & editing (lead). **Fang‐Ling Shi:** Validation (lead); Visualization (equal). **Yang Yang:** Funding acquisition (equal); Supervision (lead); Writing–review & editing (equal).

## Data Availability

The data used to support the findings of this study are available from the corresponding author upon request.
